# Dystrophin and mini-dystrophin quantification by mass spectrometry in skeletal muscle for gene therapy development in Duchenne muscular dystrophy

**DOI:** 10.1038/s41434-021-00300-7

**Published:** 2021-11-05

**Authors:** Vahid Farrokhi, Jason Walsh, Joe Palandra, Joanne Brodfuehrer, Teresa Caiazzo, Jane Owens, Michael Binks, Srividya Neelakantan, Florence Yong, Pinky Dua, Caroline Le Guiner, Hendrik Neubert

**Affiliations:** 1 Biomedicine Design, Worldwide Research & Development, Pfizer Inc, 1 Burtt Road, Andover, MA 01810 USA; 2 Biomedicine Design, Worldwide Research & Development, Pfizer Inc, 610 Main Street, Cambridge, MA 02139 USA; 3 Rare Disease Research Unit, Pfizer Worldwide Research & Development, 610 Main Street, Cambridge, MA 02139 USA; 4 Clinical Pharmacology, Early Clinical Development, Worldwide Research & Development, Pfizer Inc, 1 Portland St, Cambridge, MA 02139 USA; 5 Biostatistics, Worldwide Research & Development, Pfizer Inc, Cambridge, MA 02139 USA; 6 Early Clinical Development, Clinical Pharmacology, Pfizer R&D UK Limited, Cambridge, UK; 7 Translational Gene Therapy Laboratory, University of Nantes, INSERM UMR1089, CHU de Nantes, IRS 2 Nantes Biotech, 22 Boulevard Benoni Goulin, 44200 Nantes, France

**Keywords:** Gene expression analysis, Neurological disorders, Gene expression

## Abstract

Duchenne muscular dystrophy (DMD) is a lethal, degenerative muscle disorder caused by mutations in the *DMD* gene, leading to severe reduction or absence of the protein dystrophin. Gene therapy strategies that aim to increase expression of a functional dystrophin protein (mini-dystrophin) are under investigation. The ability to accurately quantify dystrophin/mini-dystrophin is essential in assessing the level of gene transduction. We demonstrated the validation and application of a novel peptide immunoaffinity liquid chromatography–tandem mass spectrometry (IA-LC-MS/MS) assay. Data showed that dystrophin expression in Becker muscular dystrophy and DMD tissues, normalized against the mean of non-dystrophic control tissues (*n* = 20), was 4–84.5% (mean 32%, *n* = 20) and 0.4–24.1% (mean 5%, *n* = 20), respectively. In a DMD rat model, biceps femoris tissue from dystrophin-deficient rats treated with AAV9.hCK.Hopti-Dys3978.spA, an adeno-associated virus vector containing a mini-dystrophin transgene, showed a dose-dependent increase in mini-dystrophin expression at 6 months post-dose, exceeding wildtype dystrophin levels at high doses. Validation data showed that inter- and intra-assay precision were ≤20% (≤25% at the lower limit of quantification [LLOQ]) and inter- and intra-run relative error was within ±20% (±25% at LLOQ). IA-LC-MS/MS accurately quantifies dystrophin/mini-dystrophin in human and preclinical species with sufficient sensitivity for immediate application in preclinical/clinical trials.

## Introduction

Duchenne muscular dystrophy (DMD), one of the most common X-linked recessive disorders, affects ~1 in 5000 live male births, with an estimated 20,000 children diagnosed globally each year [1, 2]. DMD is characterized by progressive muscle degeneration and weakness [3]. Diagnosed at ~4 years of age, the majority of patients are wheelchair-dependent by about age 12 years, require assisted ventilation at 20 years or older, and have a life expectancy of 20–40 years [4, 5].

DMD is caused by mutations in the *DMD* gene, resulting in the absence or reduction of the protein dystrophin [6]. Dystrophin is a large 427 kDa protein with an mRNA of 14 kb composed of 79 exons [7]. Mutations that maintain the dystrophin reading frame tend to produce a truncated, but partially functioning dystrophin protein that causes the milder form, Becker muscular dystrophy (BMD). Patients with BMD have a later onset of skeletal muscle weakness and demonstrate slower disease progression. In patients with BMD, a dystrophin level >10% relative to normal appears to be sufficient to avoid a more severe disease course [8]. Observations from patients with X-linked cardiomyopathy suggest that full-length dystrophin levels of 30% in skeletal muscle may be sufficient to prevent muscular dystrophy [9]. Although, evidence from animal models suggests the threshold to restore muscle function may be higher (>40%) [10], restoration of dystrophin in skeletal muscle represents a viable therapeutic approach.

Numerous strategies aimed at restoring dystrophin in muscle are being explored, including exon skipping, CRISPR and gene delivery using viral vectors. One promising strategy is to use adeno-associated virus (AAV) vectors [11], which have shown high-level, persistent, systemic expression in muscle cells with an apparent lack of pathogenicity [12]. A major obstacle to the use of AAV vectors is the limited packaging size that only allows for genes smaller than 4.5 kb, therefore precluding a large gene, such as dystrophin with a complementary DNA of 14 kb [13]. Stemming from observations in patients with BMD, numerous mini (or micro)-dystrophins have been developed. These highly truncated versions of dystrophin restore a certain level of function in preclinical mouse and canine models [13–16]. AAV9.hCK.Hopti-Dys3978.spA, a recombinant AAV9 capsid containing a human mini-dystrophin gene under control of a muscle-specific promoter, is one such therapy under investigation in a multicentre, open-label, non-randomized, ascending-dose study in boys aged 4 years and older with DMD (ClincialTrials.gov, NCT03362502) [17].

Evidence of a correlation of dystrophin/mini-dystrophin expression with functional outcomes would support the use of a quantified target protein for the accelerated approval of new treatments [18]. Indeed, the antisense oligonucleotides Exondys 51 (approved 2016), Vyondys 53 (approved 2019) and Amondys 45 (approved 2021) all received accelerated approval from the US Food and Drug Administration based on the surrogate endpoint of increased dystrophin in skeletal muscle as assessed by Western blot [19–21].

Traditionally, Western blots have been used to evaluate dystrophin expression in a semi-quantitative manner. However, reproducibility of Western blot results is less than optimal as they can produce a high level of inter-laboratory variation, especially with samples nearing the lower limit of quantification (LLOQ) [22]. This may partly be due to the absence of a dystrophin reference standard that can be controlled and used across all assay runs and all laboratories [23]. Other variables affecting the reproducibility of Western blots for quantification include gel overloading, membrane transfer efficiency, use of different detection antibodies, nonspecific binding of detection antibodies, different data analysis methodology and signal development [24].

The development of a specific and sensitive quantitative method to measure endogenous and mini-dystrophin expression in skeletal muscle tissue would be of substantial benefit to drug development and monitoring of treatment. To this end, liquid chromatography–tandem mass spectrometry (LC-MS/MS) has emerged as a method for human dystrophin quantification. The initial method based on sodium dodecyl sulphate (SDS) PAGE separation, in-gel digestion and LC-MS/MS of dystrophin derived tryptic peptides has detected dystrophin levels at 5% relative to normal expression in healthy individuals [25]. Subsequent studies using similar sample preparation methods greatly advanced sensitivity to achieve an LLOQ at or just below 1% of normal using 50–75 cryosections of human skeletal muscle at 10-μm thickness each [26, 27]. However, full bioanalytical validation of these methods is limited by the variability and throughput of the gel-based sample preparation procedure. Therefore, further technology advancements are needed to enable bioanalytical validation and confident use of LC-MS/MS for dystrophin quantification, using smaller sample amounts while maintaining assay sensitivity of ~1% of normal. Peptide immunoaffinity (IA) linked to LC-MS/MS is a quantitative tool that can easily be adapted to enhance sensitivity, specificity, accuracy and precision for specific protein biomarkers [28]. Initially reported for the quantification of human plasma proteins [29], peptide IA-LC-MS/MS approaches have been developed for several tissue proteins [30–34].

We aimed to develop an IA-LC-MS/MS method that could accurately quantify dystrophin and the transgene product mini-dystrophin in skeletal muscle biopsies and be utilized for translational studies in preclinical species and clinical samples without need for modification or new reagents. By using recombinant mini-dystrophin protein as a reference standard, and SILAC [stable isotope labelling by amino acids in cell culture] mini-dystrophin for normalization, the full validation of this method and complete quantification of dystrophin was possible for the first time. We used this method to examine the expression level of endogenous dystrophin in DMD, BMD and non-dystrophic control skeletal muscle tissue. Subsequently, mini-dystrophin and endogenous dystrophin was quantified using this novel methodology in a dose-finding preclinical rat study of AAV9.hCK.Hopti-Dys3978.spA.

## Materials and methods

### Muscle samples

Frozen tissue from human muscle biopsies was provided by the Iowa Wellstone Muscular Dystrophy Specialized Research Centre funded by NINDS: U54, NS053672. Core B of this centre maintains a biorepository of muscle biopsies that were originally evaluated at the University of Iowa as diagnostic biopsies. The University of Iowa institutional review board approved the Core B Repository (IRB# 200510769; original approval 16 February 2006; most recent continuing review approval was 16 July 2020).

Twenty patient samples each from non-dystrophic controls, BMD and DMD muscle were received. Mean age (range) of the patients at the time of biopsy was 10 (4–18), 8 (3–19) and 6 (3–10) years for control subjects, patients with BMD and those with DMD, respectively. Approximately 90% of samples were biopsied from quadriceps muscle. One patient biopsy was collected from the gastrocnemius muscle and the muscle source of six samples was unknown. The non-dystrophic control samples were collected from males (*n* = 12) and females (*n* = 8). All patients with BMD or DMD were male (*n* = 40). Sample preparation and assessment, tissue lysis and extraction, protein precipitation, on-filter protein digestion and positive pressure filtration, and quantification strategy are described in detail in the Supplementary methods.

### Tissue processing and sample preparation

**Fig. 1:**
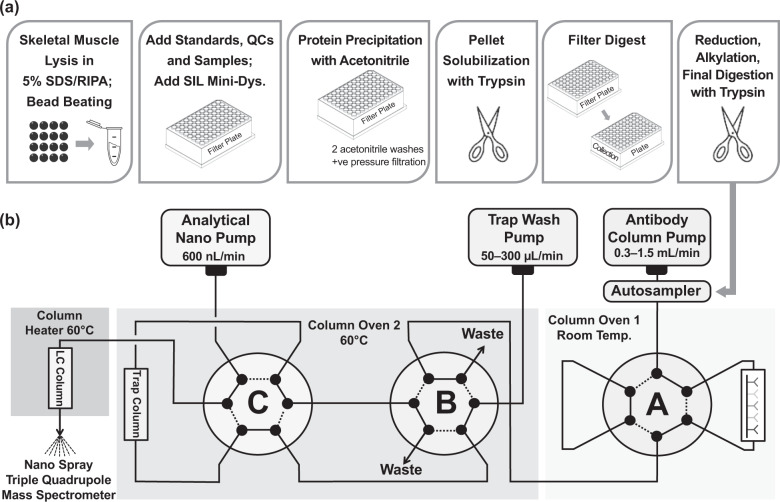
Immunoaffinity LC-MS/MS workflow and LC configuration. (**a**) Tissue processing steps and (**b**) configuration of the online immunoaffinity LC-MS/MS. LC liquid chromatography, MS/MS tandem mass spectrometry.

Tissue lysis with SDS was critical for dystrophin extraction and optimal extraction was achieved with 5% SDS in RIPA lysis buffer (Fig. 1a; Supplementary Fig. S1). Although SDS is commonly used in Western blot methods, it must be subsequently removed during sample preparation for an LC-MS/MS assay to prevent interference with downstream sample preparation steps and LC-MS/MS [35]. Protein precipitation, washing the pellets with acetonitrile and the peptide enrichment in antibody column coupled online with LC-MS/MS allowed for effective removal of SDS as well as optimal cutting temperature compound (OCT), and also served to concentrate the sample (Fig. 1a) [32]. The use of OCT in muscle tissue samples had no effect on assay performance (Supplementary Fig. S2).

### Peptide selection

Targeted dystrophin and mini-dystrophin peptide sequences were selected based on key criteria to meet the assay objectives. Briefly, specificity to mini-dystrophin or full-length dystrophin, specificity to rat dystrophin and antigenicity to enable generating specific antipeptide antibodies, formed the main criteria. From the three selected targeted peptides, LLQVAVEDR (LLQV), LEMPSSLMLEVPTHR (LEMP) and SLEGSDDAVLLQR (SLEG), LLQV is present in dystrophin and mini-dystrophin sequences. It is expressed in human, cynomolgus, rattus and mus, but not in canis species. Upon digestion, this peptide is produced in an equimolar fashion by both dystrophin and mini-dystrophin, i.e., one mole of dystrophin/mini-dystrophin produces 1 mole LLQV. Peptide LEMP is present in mini-dystrophin only and does not occur in the proteome of humans or any preclinical species. It spans a junction between a dystrophin hinge region and rod domain created by deletion of large portion of the central rods and hinges [13]. The LEMP peptide is the only viable tryptic peptide specific to mini-dystrophin. The presence of methionine residues prone to oxidation have been accounted for in the assay, given that the quantification strategy uses an internal stable isotope labelled mini-dystrophin standard which is added to each unknown sample, QC sample and calibration standard at the very beginning of the workflow. If oxidation were to occur during sample processing or analysis, both the LEMP peptide and its stable isotope labelled counterpart would be affected equally, thereby removing any potential adverse impact on accuracy. SLEG is present in human and cynomolgus dystrophin and mini-dystrophin but not canis, rattus and mus species. In a rat study of AAV9.hCK.Hopti-Dys3978.spA, as presented herein, SLEG is therefore used as a measure of mini-dystrophin expression.

### IA-LC-MS/MS assay

Similar IA-LC-MS/MS methods have been previously described [30, 31, 36, 37]. A schematic of the IA-LC-MS/MS configuration is presented in Fig. 1b (additional detail in Supplementary Table S1). Briefly, the sample is loaded and the flow through the antibody column is controlled by valve A. Peptides of interest are captured and unwanted peptides and contaminants are removed to waste (valve B), ensuring that the trap remains clean. The antibody column is washed with 25 mM ammonium formate, followed by 0.5% trifluoroacetic acid in water to elute target peptides onto the C18 trap. Chromatographic separation is subsequently achieved in the Thermo Easy Spray PepMap C18 at a flow rate of 0.6 µl/min with acetonitrile/formic acid/water buffers and at an injection volume of 80 µl. Analysis of human samples used a single antipeptide Ab column holding two antipeptide antibodies against LLQV and LEMP peptides. Analysis of the dystrophin-deficient (DMD^*mdx*^) rat samples utilized antipeptide antibodies against peptides LLQV and SLEG as LEMP reagents were not available at the time of analysis.

All custom antipeptide antibodies (immunoglobulin G) were generated by Cambridge Research Biochemicals and the antipeptide antibody columns were prepared in-house using IDEX Biosafe Column System (2.1 mm × 30 mm × 2.0 µm). The antibody solution was prepared so as to include anti-LLQV and anti-LEMP antibodies ranging from 0.30–0.40 mg (for anti-LLQV) and 0.8–1.0 mg (for anti-LEMP) per antibody column. Anti-SLEG was used at 0.5 mg per antibody column. The trap column and nano LC were maintained at a temperature of 60 °C and eluent from the antipeptide antibody columns was collected on a µ-Precolumn Cartridge fitted with a PepMap™ 100 C18 (ThermoFisher) with 5-µm particle size, 100 Å pore size, 300 µm diameter, 5-mm length. Chromatographic separation was achieved using Easy Spray PepMap C18 column (75 µm × 15 cm).

The eluate from nanoflow chromatography was introduced into Easy Spray Ionization Source (ThermoFisher) at 60 °C with a coupling spray voltage of 3000 V and a collision gas pressure of 1.5 mTorr. Detection of peptides was performed on a Quantiva Triple Quadrupole MS (ThermoFisher) by multiple reaction monitoring in positive ion mode. MS parameter settings and multiple reaction monitoring transitions are provided in Supplementary Table S2. Echo transition summing was used to enhance signal-to-noise and sensitivity of the assay for each peptide. Major precursor ions to fragment transitions were scanned multiple times during each multiple reaction monitoring cycle. Transitions, including multiple product ions, were then combined, which generated a summed, quantifiable area under the concentration–time curve. MS acquisition time was ~14.5 min, with expected retention times of 11.1 ± 1.5 and 12.1 ± 1.5 min for LLQV and LEMP, respectively.

### Method validation

Validation samples were comprised of both a normal lysate pool, representing endogenous dystrophin prepared from 20 non-dystrophic, paediatric subjects, and DMD skeletal muscle lysate void of dystrophin spiked with mini-dystrophin at the four quality control (QC) levels of 50, 200, 800 and 2400 fmol/ml mini-dystrophin. Intra- and inter-assay precision were evaluated against acceptance criteria: overall precision (% coefficient of variation [CV]) and accuracy (% relative error [RE]) ≤ 20.0% (≤25% at LLOQ) for QC samples (low, medium 1, medium 2, high). Stability studies assessed the lysate bench top stability, 72 h auto-injector stability, 7-day processed sample stability and two cycles of freeze–thaw at −70 °C. The normal lysate pool, meanwhile, was assigned a concentration during the accuracy and precision portion of the validation.

### Rat DMD^*mdx*^ model

Endogenous rat dystrophin and AAV-mediated expression of mini-dystrophin were quantified in bicep femoris muscle samples obtained from DMD^*mdx*^ rats [38], after a single intravenous administration of AAV9.hCK.Hopti-Dys3978.spA in a dose-finding efficacy study (data on file, Pfizer). Muscle samples were harvested 3 or 6 months post-administration from wild type (WT), vehicle controls and four dose groups of 1E + 13, 3E + 13, 1E + 14 and 3E + 14 vg/kg of AAV9.hCK.Hopti-Dys3978.spA. Peptide LLQV, which is conserved across humans and rats, detects total dystrophin (endogenous dystrophin and mini-dystrophin), and SLEG, which is human-specific sequence to detect mini-dystrophin only, were quantified using IA-LC-MS/MS.

## Results

### Quantification of mini-dystrophin and dystrophin protein in skeletal muscle from DMD^*mdx*^ rats treated with an AAV9-mini-dystrophin candidate gene therapy

**Fig. 2:**
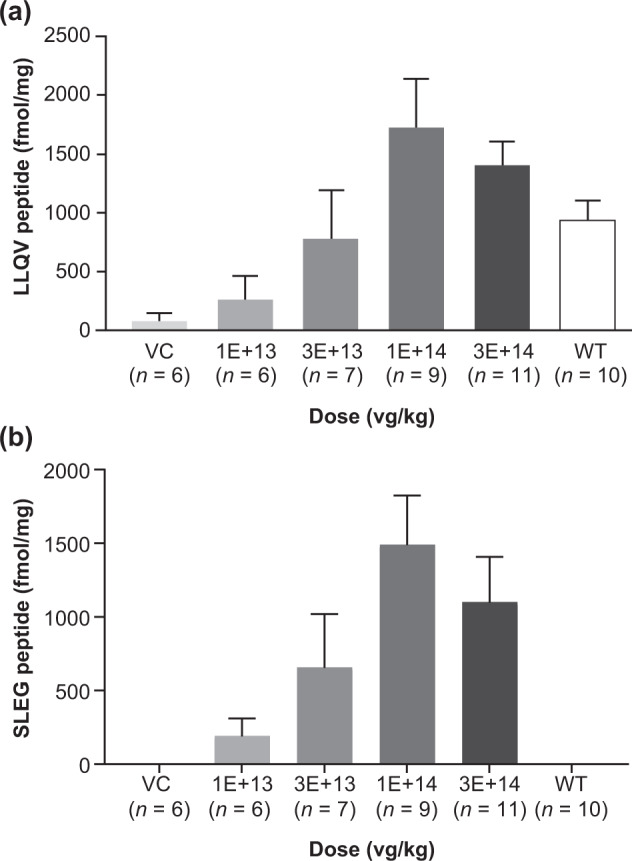
Dystrophin/mini-dystrophin expression in DMD^*mdx*^ rats. (**a**) Total dystrophin expression based on peptide LLQV and (**b**) mini-dystrophin based on peptide SLEG in biceps femoris of wild type and DMD^*mdx*^ rats for vehicle control, and four AAV9.hCK.Hopti-Dys3978.spA dose groups at 6 months post-dose. DMD^*mdx*^ dystrophin-deficient Duchenne muscular dystrophy, LLQV LLQVAVEDR, SLEG SLEGSDDAVLLQR, VC vehicle control, WT wild type.

IA-LC-MS/MS analysis of biceps femoris tissue samples from DMD^*mdx*^ rats treated with increasing doses of AAV9.hCK.Hopti-Dys3978.spA at 3 and 6 month post-dose, showed a dose-dependent increase in mini-dystrophin protein expression as measured by two peptides, LLQV and SLEG. Expression of LLQV (total dystrophin) and SLEG (mini-dystrophin) were comparable, with a maximum total dystrophin observed in the 1E + 14 vg/kg dose group (Fig. 2a, b). Compared with WT rats, mean molar levels of mini-dystrophin protein in the 1E + 14 vg/kg dose group surpassed mean normal levels of dystrophin in WT rats by up to 150%. The concentration of revertant fibre dystrophin in the control group of DMD^*mdx*^ rats not receiving the gene therapy vector was 7.4–10.4% of dystrophin mean expression in the WT rats.

### IA-LC-MS/MS assay validation

Peptides LLQV and LEMP were successfully validated for the analysis of dystrophin and mini-dystrophin in human skeletal muscle sample lysates for the concentration range 20.0–3333 fmol/ml (=pM). Inter- and intra-assay precision were ≤20% (≤25%) at the LLOQ and inter- and intra-run RE was within ±20% (±25% at LLOQ) (Table 1). The normal lysate pool was successfully assigned both an endogenous dystrophin concentration of 1470 fmol/ml and a total protein concentration of 0.490 mg/ml determined by bicinchoninic acid (BCA) assay.

**Table 1:** Summary of inter- and intra-assay accuracy and precision based on peptides LLQV and LEMP from five accuracy and precision runs during validation.

	Precision (%CV)		Accuracy (%RE)	
Peptide	Intra-assay	Inter-assay	Intra-assay	Inter-assay
LLQV	3.7 to 23.4	7.8 to 17.9	–10.0 to 9.0	–5.0 to 8.5
LEMP	2.7 to 22.5	9.1 to 20.6	–12.8 to 18.2	–8.0 to 12.5

Intra- and inter-run %CV was ≤20% (except ≤25% at QCLOQ) and intra and inter-run %RE was within ±20% (except ±25% at QCLOQ).

*CV* coefficient of variation, *QCLOQ* limit of quantification quality control sample, *RE* relative error.

**Fig. 3:**
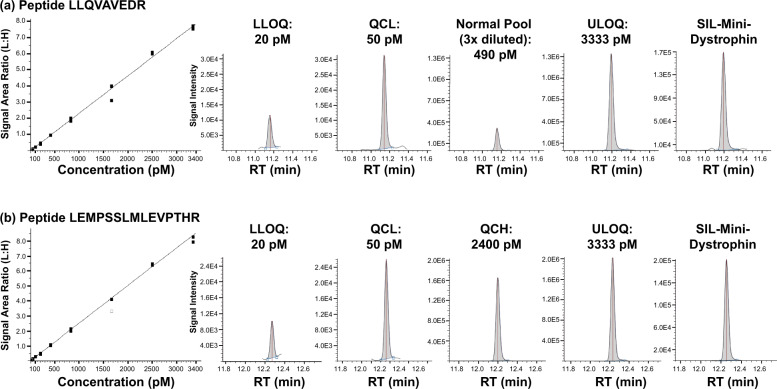
Calibration curves and chromatograms. Representative calibration curves and peptide extracted ion chromatograms for (**a**) LLQV and (**b**) LEMP peptides. LLOQ lower limit of quantification, LLQV LLQVAVEDR, LEMP LEMPSSLMLEVPTHR, QCH high quality control, QCL low quality control, RT retention time, SIL stable isotope labelled (peptide), ULOQ upper limit of quantification.

Back-calculated mini-dystrophin calibration standards for LLQV and LEMP peptides are presented in Supplementary Table S3. Representative calibration curves and ion chromatograms for peptides LLQV and LEMP are shown in Fig. 3.

Processed sample stability was established for up to 1 week at −70 °C (Supplementary Table S4) and autosampler stability established for up to 72 h. The normal lysate was found to be stable over a 30-week testing period following storage at −70 °C (Supplementary Table S5) and up to three cycles of freezing and thawing (Supplementary Table S6). Assay sensitivity was maintained, confirming the stability of the calibrator.

### Dystrophin expression in normal, BMD and DMD muscles

**Fig. 4:**
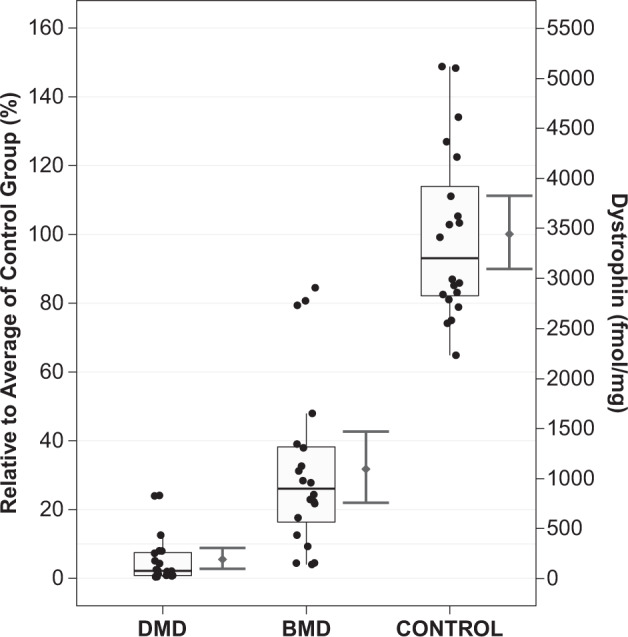
Dystrophin expression in normal/BMD/DMD human skeletal muscle. Relative concentration of dystrophin is displayed on the left y-axis and absolute concentration of dystrophin (fmol/mg total protein) on the right y-axis. The lower and upper hinges of the boxplot correspond to the first and third quartiles (the 25th and 75th percentiles); the upper whisker extends from the hinge to the largest value no further than 1.5*IQR from the hinge, and the converse is the case for the lower whiskers. Data beyond the end of the whiskers are outliers and are plotted individually. Bars are the 95% confidence intervals for the mean in each group (diamonds) based on bootstrap method [43, 44]. BMD Becker muscular dystrophy, DMD Duchenne muscular dystrophy, IQR interquartile range, LLQV LLQVAVEDR.

Twenty skeletal muscle biopsy samples each from paediatric, non-dystrophic subjects and patients with BMD or DMD were used for dystrophin expression analysis. Individual results from the dystrophin and total protein analysis in the lysate are available in Supplementary Table S7 and the summary statistics are presented in Table 2. Control-tissue dystrophin expression range was 65–149% of the control mean expression (100%, median 93%; 3440 fmol/mg protein) based on the LLQV peptide (Fig. 4). In BMD muscle, the expression range was 4–84.5% of normal mean (mean 32%, median 26%; 1089 fmol/mg protein) and in DMD muscle, the expression range was 0.4–24.1% of normal mean (mean 5%, median 2%; 186 fmol/mg protein), with no dystrophin quantifiable in 7 of 20 DMD samples. In a preliminary study prior to assay validation, we conducted LLQV and SLEG analysis on similar, but slightly different DMD/BMD/CTRL human skeletal muscle biopsies. The LLQV and SLEG peptides were in very good agreement for reporting dystrophin levels in all sample types supporting the selection of LLQV as a surrogate for dystrophin in this assay. The correlation plot between LLQV and SLEG peptides is presented in Supplementary Fig. S3. The LLOQ of the LC-MS assay for dystrophin in the tissue lysate was 20.0 fmol/ml; however, the normalized tissue LLOQ of dystrophin in fmol/mg of total protein depends on the amount of tissue or total protein used and is derived by dividing the lysate dystrophin LLOQ (20.0 fmol/ml) by the total protein concentration in lysate (mg/ml). For all below limit of quantification results, dystrophin lysate concentrations were imputed as 0.5*LLOQ for the summary statistics and graphical presentation. One patient who was diagnosed with DMD had an in-frame mutation (ex 3-30 del), generally characteristic of BMD, with a 24.1% expression level. Importantly, there was no overlap in mean (95% confidence interval) dystrophin expression between DMD and non-dystrophic muscle (Fig. 4).

**Table 2:** Summary statistics of the tissue dystrophin concentration based on peptide LLQV.

Cohort	*n*	Age (years)		Tissue dystrophin concentration (fmol/mg total protein)			Relative to average of control group (%)		
		Median (Min, Max)	Mean ± SD	Median (Min, Max)	Mean ± SD	Bootstrap 95% CI for the Mean^a^	Median (Min, Max)	Mean ± SD	Bootstrap 95% CI for the Mean^a^
DMD	20	6 (3, 10)	6 ± 1.8	74.8 (14.5, 829.2)	185.9 ± 247.0	(91, 299)	2.2 (0.4, 24.1)	5.4 ± 7.2	(3, 9)
BMD	20	8 (3, 19)	8 ± 4.1	897.2 (138.9, 2906.7)	1089.3 ± 845.7	(753, 1465)	26.1 (4.0, 84.5)	31.7 ± 24.6	(22, 43)
CTRL	20	9 (4, 18)	10 ± 4.3	3201.4 (2231.4, 5118.9)	3440.2 ± 857.2	(3091, 3824)	93.1 (64.9, 148.8)	100 ± 24.9	(90, 111)

^a^Random number generator state (random seed) was set at 2020 in R version 3.5.0 [45].

*BMD* Becker muscular dystrophy, *CI* confidence interval, *CTRL* control, *DMD* Duchenne muscular dystrophy, *min* *max* minimum, maximum, *SD* standard deviation.

Representative LLQV peptide extracted ion chromatograms from age-matched healthy, BMD and DMD muscle samples are shown in Supplementary Fig. S4. As expected, peptide LEMP was not detected in any of the samples.

There was no apparent difference in dystrophin expression between healthy male and female controls (Supplementary Fig. S5a). Although mean total protein concentrations appeared to be consistent across healthy, BMD and DMD muscle samples, the observed variability is a consequence of varying sizes of the tissue blocks from which the 10-µm sections were cut (Supplementary Fig. S5b).

## Discussion

The accurate quantification of low abundance proteins, such as dystrophin, is challenging but essential for the development and validation of gene therapies aimed at restoring cellular dystrophin. Herein we described a peptide IA-LC-MS/MS method of high accuracy, precision, selectivity and sensitivity, with application in both human clinical trials and preclinical studies for the quantification of dystrophin and mini-dystrophin. This method has several advantages over traditional methods such as Western blots, which can be subject to high levels of variation and limited sensitivity [22]. In support of DMD gene therapy development, Western blot assays are used to measure the expression of mini (or micro)-dystrophin in skeletal muscle tissue from patients receiving treatment relative to full-length, endogenous dystrophin measured alongside a non-dystrophic skeletal muscle sample pool. The intensity of bands for the transgene product, i.e., the truncated dystrophin, are compared with those of the full-length, endogenous dystrophin. However, such methods do not account for differences in how the detection anti-protein antibody binds the different dystrophin forms, nor any difference between the proteins, for example in terms of sequence or size. It is not known how these variables impact the Western blot signal and therefore the percent normal calculation. An assessment of the impact of these variables has not yet been reported but is critical to understand any measurement bias using these particular Western blot methods. Reliable relative quantification of mini (micro)-dystrophin expression analysis by Western blot would require both truncated and full-length dystrophin reference standards.

The use of antipeptide antibodies in the assay described herein eliminates many of the challenges associated with anti-protein antibodies, including a lack of capture efficiency and protein denaturation during extraction [32, 39]. In contrast to Western blot methods, our method allows a large number of samples to be run at once (96 total; 60 unknown samples), including calibrants and QCs, eliminating the need for multiple assays. As the use of OCT in muscle tissue samples had no effect on assay performance, sections for LC-MS dystrophin quantification and tissue staining by immunofluorescence, for dystrophin localization, can be taken from the same tissue block. The ability to analyse adjacent sections from the same tissue block with these two methods may improve the reliability of data interpretation. Furthermore, the use of SILAC mini-dystrophin as an internal standard that was spiked into every sample including calibrations standards, QCs and unknowns at the beginning of the workflow, ensures high precision by compensating for any variation that may occur during sample preparation. High isotopic purity of the SILAC mini-dystrophin to confirm its suitability as an internal standard was supported by the absence of a LLQV or LEMP signal in the control blanks, as well as some of the DMD samples with no detectable dystrophin levels.

A notable benefit of our method is the ability to use it in both preclinical and clinical studies without the need for modification or new reagents. Peptide LLQV is present in dystrophin and mini-dystrophin, several animal species commonly used in preclinical investigations and is produced in an equimolar fashion upon digestion, by both dystrophin and mini-dystrophin. It is therefore viable for use in clinical and preclinical investigations as a combined molar measure of dystrophin and mini-dystrophin, overcoming the challenges associated with Western blot-based percent normal calculations. To this end, we successfully demonstrated the applicability of this method in a preclinical DMD^*mdx*^ rat study, without the need for methodological changes, by showing a dose-dependent expression of mini-dystrophin protein several months post-administration of AAV9.hCK.Hopti-Dys3978.spA.

Our method demonstrated high performance, particularly in terms of %RE and precision, even for low-level detection of dystrophin and mini-dystrophin. The reproducibility of our method compares favourably with that of other published techniques for the quantification of dystrophin, including capillary Western immunoassays [40] and high-throughput immunofluorescence [41], and exceeds that reported for traditional Western blots [22].

This assay allowed the quantification of dystrophin at the low femtomole per millilitre level and was able to detect dystrophin in DMD muscle samples as low as ~1% relative to healthy human muscle. This sensitivity is partly enabled due to the IA-LC-MS/MS configuration. A high flow rate at the antibody column pump and the large binding capacity of the antibody columns allowed a relatively large volume of the processed sample to be loaded rapidly while taking advantage of the sensitivity gains provided by the analytical nanoflow chromatography and nanospray ionization on the MS. This is important for the detection of low abundance proteins, such as dystrophin. A similar approach was successfully employed in the quantification of the human neonatal Fc receptor in transgenic mice and human tissues [30, 31].

To evaluate the response to a given treatment, pre-treatment dystrophin levels need to be accurately quantified. As most patients with DMD produce trace amounts of dystrophin, or have revertant fibres, methods to quantify dystrophin must be sensitive enough to differentiate between low levels of expression. Our assay allowed the quantification of dystrophin at the low femtomole per millilitre level, and was therefore able to detect very small differences in dystrophin expression and sensitive enough to detect DMD revertant fibres. Our results reflect the heterogeneity of dystrophin expression seen in patients with BMD or DMD and were relatively similar to those reported using a capillary Western immunoassay (BMD, 10–90%; DMD, 0.7–7%) [40]. In the same study, dystrophin expression in healthy human tissue ranged from 49–149% or 32–173%, depending on the antibody used, highlighting the importance of antibody selection in Western blot methods and consequently the potential for high variability. With the average levels of dystrophin in patients with DMD measured as 5%, our results are also consistent with previous reports that expression of endogenous dystrophin at levels >10% of those in healthy tissue, may be sufficient to protect from a more severe disease phenotype [8]. Similarly, a mean of 32% dystrophin expression in patients with BMD was consistent with previous reports suggesting ~30% of native dystrophin may be sufficient to reduce muscle weakness [9].

Overall, the IA-LC-MS/MS method described herein accurately quantifies dystrophin/mini-dystrophin in human and preclinical species with sufficient sensitivity. This IA-LC-MS/MS method is immediately applicable to clinical trials in gene therapy and would be invaluable in establishing the link between mini-dystrophin transgene protein expression and function, as well as providing guidance for dosing and method of administration. The adaptability of the assay for different protein biomarkers [29, 31–33, 36, 37, 42] and the ability to develop multi-analyte panels broadens the utility of the method.

## Supplementary information

Supplementary Material

## Data Availability

Upon request, and subject to certain criteria, conditions and exceptions (see https://www.pfizer.com/science/clinical-trials/trial-data-and-results for more information), Pfizer will provide access to individual de-identified participant data from Pfizer-sponsored global interventional clinical studies conducted for medicines, vaccines and medical devices (1) for indications that have been approved in the US and/or EU or (2) in programmes that have been terminated (i.e., development for all indications has been discontinued). Pfizer will also consider requests for the protocol, data dictionary, and statistical analysis plan. Data may be requested from Pfizer trials 24 months after study completion. The de-identified participant data will be made available to researchers whose proposals meet the research criteria and other conditions, and for which an exception does not apply, via a secure portal. To gain access, data requestors must enter into a data access agreement with Pfizer.
