# Supplementary material for: Dystrophin and mini-dystrophin quantification by mass spectrometry in skeletal muscle for gene therapy development in Duchenne muscular dystrophy
Source: Gene Ther. 2021 Nov 5;29(10-11):608–15. doi: 10.1038/s41434-021-00300-7 (PMC9068826; doi:10.1038/s41434-021-00300-7)
Supplement: Supplementary file 1 — Supplementary Material [file 41434_2021_300_MOESM1_ESM.docx]

**Supplementary Information**

**Dystrophin and Mini-Dystrophin Quantification by Mass Spectrometry in Skeletal Muscle for Gene Therapy Development in Duchenne Muscular Dystrophy**

**Contents**

[Supplementary Methods 2](#_Toc84322201)

[Sample Preparation and Assessment 2](#_Toc84322202)

[Tissue Lysis and Extraction 2](#_Toc84322203)

[Protein Precipitation 2](#_Toc84322204)

[On-filter Protein Digestion and Positive-Pressure Filtration 3](#_Toc84322205)

[Quantification Strategy 3](#_Toc84322206)

[References 4](#_Toc84322207)

[Supplementary Table S1. Liquid chromatography system description. 5](#_Toc84322208)

[Supplementary Table S2. (a) Mass spectrometer parameters and (b) MRM transitions 6](#_Toc84322209)

[Supplementary Table S3. Calibration curve performance for (a) LLQV and (b) LEMP peptides. 8](#_Toc84322210)

[Supplementary Table S4. One-week freezer stability (–70⁰C) of endogenous dystrophin and mini-dystrophin in skeletal muscle lysate for (a) LLQV and (b) LEMP. 10](#_Toc84322211)

[Supplementary Table S5. Normal lysate stability over a 30-week testing period following storage at –70°C. 11](#_Toc84322212)

[Supplementary Table S6. Stability of mini-dystrophin in skeletal muscle lysate after cycles of freezing and thawing for (a) LLQV and (b) LEMP. 12](#_Toc84322213)

[Supplementary Table S7. Dystrophin and total protein analysis for (a) control, non-dystrophic subjects, (b) BMD patients and (c) DMD patients. 13](#_Toc84322214)

[Supplementary Table S8. SILAC mini-dystrophin amino acid sequence. All leucine residues were labelled with 13C. Peptide sequences of interest are underlined. Amino acids in bold are labelled on target peptide sequences. 16](#_Toc84322215)

[Supplementary Figures 18](#_Toc84322216)

[Supplementary Fig. S1. 18](#_Toc84322217)

[Supplementary Fig. S2. 19](#_Toc84322218)

[Supplementary Fig. S3. 20](#_Toc84322219)

[Supplementary Fig. S4. 21](#_Toc84322220)

[Supplementary Fig. S5. 22](#_Toc84322221)

# Supplementary Methods

## Sample Preparation and Assessment

Study samples were received as cryosections (15 slices of 10 µm) in a moderate amount of optimal cutting temperature compound (OCT) and stored at –70°C. Method workflow is summarized in Fig. 1a. Samples with excessive OCT had 1 ml of ice cold 70% ethanol added and were vortexed 5×5 s (medium speed) and subsequently centrifuged at 14,000 rpm at 4°C for 10 min and the supernatant discarded and air dried at ambient temperature for 5 min. Preclinical samples were weighed prior to tissue lysis.

The effect of sodium dodecyl sulfate (SDS) in the lysis buffer on percentage dystrophin extraction was evaluated by comparing RIPA buffer (0.1% SDS) with TER-I (no SDS); RIPA buffer with 5%, 2%, and 0.1% SDS; and re-extraction from debris relative to 5% SDS.

## Tissue Lysis and Extraction

Approximately 10 beads of a 0.9–2.0-mm blend of stainless steel beads (NextAdvance™, Averill Park, NY, USA) were added to 500 µl lysis buffer consisting of RIPA buffer with 5% SDS (Fisher Scientific International, Hampton, NH, USA) and 1× HALT Protease Inhibitor Cocktail (ThermoFisher Scientific, Waltham, MA, USA) at 100 µl per 10 ml lysis buffer. Tissues were homogenized at room temperature using the Bullet Blender® (Next Advance) for ~10 min. Lysates were clarified by centrifugation at room temperature and 14,000 rpm for 20 min. Homogenization and centrifugation was repeated using a second aliquot of 500 µl per 10 ml lysis buffer. A 25 µl aliquot of each sample retained for the bicinchoninic assay (BCA) protein determination assay.

Healthy and Duchenne muscular dystrophy (DMD) muscle lysates were diluted at a ratio of 1.5-mg tissue mass to 1000-µl lysis buffer, with the volume of lysis buffer adjusted to the mass of the sample cut (~0.2 mg minimum). A 25 µl aliquot of each sample was stored at room temperature in a clean Eppendorf tube (Hauppauge, NY, USA) until the BCA total protein assay could be performed. The 120 µl aliquot of study samples, calibration curve and quality control (QC) samples were added to the appropriate wells on the filter plate.

Stable isotope-labelling by amino acids in cell culture (SILAC) mini-dystrophin (KempBio, Frederick, MD, USA) was used as the internal standard. The SILAC mini-dystrophin amino acid sequence is presented in Table S8.

SILAC mini-dystrophin working solution was prepared by diluting 400 µl of the 40 pmol/ml stock with 3600 µl of surrogate matrix. The surrogate matrix was prepared by adding serum to the lysis buffer at a final concentration of 0.7%. SILAC mini-dystrophin working solution was added to each sample (20 µl of 2,000 fmol/ml) and incubated for 4–6 min at room temperature.

## Protein Precipitation

A 120 µl aliquot of each sample was precipitated in 800 µl acetonitrile at room temperature for 25–30 min with gentle mixing at 300 rpm in the Fisher Isotemp Shake Touch. The supernatant was removed using positive-pressure manifold connected to nitrogen gas and the flow through discarded (pressure ~20 psi). Protein pellets were washed in 1 ml acetonitrile per sample at room temperature and the wash filtered out by positive-pressure manifold. This step was repeated and the filter air dried. Protein pellets were solubilized in 120 µl of buffer comprised of 80% PBS, 10% 8M urea, 10% acetonitrile and 50 μg/ml of tosyl phenylalanyl chloromethyl ketone (TPCK)–treated trypsin (6.0 μg per well). The filter plate was sealed and incubated at 37°C in the Fisher Isotemp Shake Touch at 900 rpm for 12–18 h.

## On-filter Protein Digestion and Positive-Pressure Filtration

The plate was briefly spun down (≤400 rpm for 15 s) and pellets further solubilized by adding 30 µl of TPCK-trypsin prepared in PBS (1.5 µg per well) to each sample, incubating in the Heratherm incubator at 37°C with shaking at 900 rpm for 2.5–3.5 h. The plate was briefly spun down and using positive-pressure manifold and the digested mixture was filtered directly into a 1.5 ml 96-deep well collection plate placed below the filter plate. An initial pressure of 20 psi was used and adjusted accordingly (not to exceed 25 psi). The filter plate was washed using 100 µl of 15% acetonitrile and positive-pressure filtration. Disulfide reduction was carried out at 60°C for 50–70 min by adding 10 µl of freshly prepared 150 mM dithiothreitol to each sample, followed by alkylation at room temperature, in the dark, for 50–70 min with 10 µl of 300 mM iodoacetamide. Samples were subsequently digested at 37°C for ≥3 h by adding 10 µl of 100 µg/ml LysC-trypsin. Samples were injected onto a high-performance liquid chromosome system (Dionex UltiMate™ 3000; ThermoFisher).

## Quantification Strategy

Dystrophin expression was calculated as a percentage of dystrophin expression in the healthy sample pool. Mini-dystrophin (fmol/ml) was calculated as a percentage of total protein (mg) and back-calculated against a mini-dystrophin standard curve. Calibrant standards were freshly prepared in 0.7% human serum in lysis buffer. Mini-dystrophin was spiked into DMD lysate prior to protein extraction and digestion. Mini-dystrophin calibrant concentrations were 3,333, 2,500, 1,667, 833, 407, 208, 104, 52.1, 26.0, 20.0 and 0 fmol/ml. Duplicate calibration standards were included in each 96-well plate.

Total protein QC samples were freshly prepared and measured in replicates of four with spiking BSA into DMD tissue lysate at the following concentrations 1,000, 500, and 225 µg/ml. Peptide concentrations from healthy, BMD and DMD lysate samples were normalized to total protein content, as determined by a photometric BCA assay (Pierce™ BCA Protein Analysis kit; ThermoFisher). Reported mean (95% confidence interval) dystrophin expression in healthy, BMD, and DMD muscle was based on 10,000 bootstrap samples of size 20 [1, 2].

All liquid chromatography–tandem mass spectrometry data were generated by TraceFinder™ General Quan v. 4.2 (ThermoFisher). Peaks were identified by retention time using the associated raw data file from a calibration curve sample (single peak detection using the ICIS detection algorithm, default settings). Peaks areas from all associated transition summing were compiled and the area under the concentration–time curve (AUC) imported to Watson LIMS. AUC data were analysed for concentration based on back-calculated data from the calibration curve. The calibration curve was fitted with a linear regression model and 1/×2 weighting.

## References

1. R Core Team. R: a language and environment for statistical computing. 2014. http://www.R-project.org (accessed 8 March 2021).

2. Neubert H, Gale J, Muirhead D. Online high-flow peptide immunoaffinity enrichment and nanoflow LC-MS/MS: assay development for total salivary pepsin/pepsinogen. Clin Chem. 2010: 56: 1413-1423. doi: 10.1373/clinchem.2010.144576.

# Supplementary Table S1. Liquid chromatography system description.

| **Micro Pump:**  **Mobile Phases and Program** | **A: 25mM Ammonium Formate**  **B: 0.50 % Trifluoroacetic Acid in Water**  **C: 0.75 % Formic Acid, 3% Isopropanol in Water**   \| **Retention**  **Time (min)** \| **Flow Rate (ml/min)** \| **%A** \| **%B** \| **%C** \| \| --- \| --- \| --- \| --- \| --- \| \| 0.00 \| 0.600 \| 100 \| 0 \| 0 \| \| 3.00 \| 0.600 \| 100 \| 0 \| 0 \| \| 3.00 \| 0.300 \| 0 \| 100 \| 0 \| \| 6.00 \| 0.300 \| 0 \| 100 \| 0 \| \| 6.00 \| 1.00 \| 0 \| 0 \| 100 \| \| 9.50 \| 1.00 \| 0 \| 0 \| 100 \| \| 9.50 \| 1.00 \| 100 \| 0 \| 0 \| \| 12.00 \| 1.00 \| 100 \| 0 \| 0 \| \| 12.50 \| 0.100 \| 100 \| 0 \| 0 \| |
| --- | --- | --- | --- | --- | --- | --- | --- | --- | --- | --- | --- | --- | --- | --- | --- | --- | --- | --- | --- | --- | --- | --- | --- | --- | --- | --- | --- | --- | --- | --- | --- | --- | --- | --- | --- | --- | --- | --- | --- | --- | --- | --- | --- | --- | --- | --- | --- | --- | --- | --- | --- |
| **Loading Pump:**  **Mobile Phases and Program** | **A: 0.1% Trifluoroacetic Acid in Water**  **C: 90/10/0.1% Acetonitrile/Water/Formic Acid**   \| **Retention**  **Time (min)** \| **Flow Rate (ml/min)** \| **%A** \| **%C** \| \| --- \| --- \| --- \| --- \| \| 0.00 \| 0.250 \| 15 \| 85 \| \| 0.05 \| 0.250 \| 15 \| 85 \| \| 1.00 \| 0.250 \| 100 \| 0 \| \| 2.75 \| 0.250 \| 100 \| 0 \| \| 3.00 \| 0.050 \| 100 \| 0 \| \| 3.25 \| 0.050 \| 70 \| 30 \| \| 9.00 \| 0.050 \| 70 \| 30 \| \| 12.10 \| 0.050 \| 70 \| 30 \| \| 12.15 \| 0.250 \| 70 \| 30 \| \| 12.50 \| 0.250 \| 15 \| 85 \| |
| **Nano (NC) Pump:**  **Mobile Phases and Program** | **A: 98/2/0.1% Water/Acetonitrile/Formic Acid**  **B: 90/10/0.1% Acetonitrile/Water/Formic Acid**   \| **Retention Time (min)** \| **Flow rate (µl/min)** \| **%A** \| **%B** \| \| --- \| --- \| --- \| --- \| \| 0.00 \| 0.600 \| 15 \| 85 \| \| 1.00 \| 0.600 \| 15 \| 85 \| \| 2.00 \| 0.600 \| 98 \| 2 \| \| 6.00 \| 0.600 \| 98 \| 2 \| \| 11.00 \| 0.600 \| 65 \| 35 \| \| 11.50 \| 0.600 \| 65 \| 35 \| \| 11.50 \| 0.600 \| 15 \| 85 \| |
| **Valve Switching**  **(Column Oven NCS-3500RSC)** | \| **Retention  Time (min)** \| **Valve 2** \| **Valve 2** \| \| --- \| --- \| --- \| \| Initial \| 1_2 \| 6_1 \| \| 3.25 \| 6_1 \| 6_1 \| \| 6.00 \| 1_2 \| 1_2 \| \| 12.00 \| 1_2 \| 6_1 \| |
| **Valve Switching (Column Oven TCC-3200RS)** | \| **Retention  Time (min)** \| **Valve 1** \| \| --- \| --- \| \| Initial \| 6_1 \| |

# Supplementary Table S2. (a) Mass spectrometer parameters and (b) MRM transitions

**(a) Mass spectrometer parameters**

| MS system | Thermo Quantiva Triple Quadrupole |
| --- | --- |
| Ion source | Thermo Fisher Scientific Easy Spray Ionization Source |
| LC coupling spray voltage, V | 3000 |
| Heated capillary temperature, °C | 300 |
| Collision gas pressure, mTorr | 1.5 |
| MS acquisition time, min | 14.5 |
| Polarity | Positive |
| Data type | Centroid |
| Q1, FWHM | 0.70 |
| Q3, FWHM | 0.70 |
| Scan width, m/z | 0.010 |

**(b) MRM transitions**

| **Compound** | **Start Time**  **(min)** | **End Time**  **(min)** | **Polarity** | **Precursor  (m/z)** | **Product  (m/z)** | **Fragment Type/Number** | **Collision Energy**  **(V)** | **Dwell Time**  **(ms)** |
| --- | --- | --- | --- | --- | --- | --- | --- | --- |
| LLQV | 8 | 11.6 | Positive | 521.798 | 589.294 | [y5] | 19.4 | 60 |
| LLQV | 8 | 11.6 | Positive | 521.798 | 688.362 | [y6] | 19.4 | 60 |
| LLQV | 8 | 11.6 | Positive | 521.798 | 816.421 | [y7] | 19.4 | 60 |
| LLQV (H) | 8 | 11.6 | Positive | 527.818 | 589.294 | [y5] | 19.5 | 60 |
| LLQV (H) | 8 | 11.6 | Positive | 527.818 | 688.362 | [y6] | 19.5 | 60 |
| LLQV (H) | 8 | 11.6 | Positive | 527.818 | 816.421 | [y7] | 19.5 | 60 |
| LEMP | 11.6 | 14.5 | Positive | 580.632 | 609.335 | [y5] | 26.3 | 20 |
| LEMP | 11.6 | 14.5 | Positive | 580.632 | 609.339 | [y5] | 26.3 | 20 |
| LEMP | 11.6 | 14.5 | Positive | 580.632 | 609.343 | [y5] | 26.3 | 20 |
| LEMP | 11.6 | 14.5 | Positive | 580.632 | 609.347 | [y5] | 26.3 | 20 |
| LEMP | 11.6 | 14.5 | Positive | 580.632 | 609.351 | [y5] | 26.3 | 20 |
| LEMP | 11.6 | 14.5 | Positive | 580.632 | 609.355 | [y5] | 26.3 | 20 |
| LEMP | 11.6 | 14.5 | Positive | 580.632 | 609.359 | [y5] | 26.3 | 20 |
| LEMP | 11.6 | 14.5 | Positive | 580.632 | 738.385 | [y6] | 26.3 | 20 |
| LEMP | 11.6 | 14.5 | Positive | 580.632 | 738.389 | [y6] | 26.3 | 20 |
| LEMP | 11.6 | 14.5 | Positive | 580.632 | 738.393 | [y6] | 26.3 | 20 |
| LEMP (H) | 11.6 | 14.5 | Positive | 586.652 | 609.337 | [y5] | 26.3 | 20 |
| LEMP (H) | 11.6 | 14.5 | Positive | 586.652 | 609.341 | [y5] | 26.3 | 20 |
| LEMP (H) | 11.6 | 14.5 | Positive | 586.652 | 609.345 | [y5] | 26.3 | 20 |
| LEMP (H) | 11.6 | 14.5 | Positive | 586.652 | 609.349 | [y5] | 26.3 | 20 |
| LEMP (H) | 11.6 | 14.5 | Positive | 586.652 | 609.353 | [y5] | 26.3 | 20 |
| LEMP (H) | 11.6 | 14.5 | Positive | 586.652 | 609.357 | [y5] | 26.3 | 20 |
| LEMP (H) | 11.6 | 14.5 | Positive | 586.652 | 609.361 | [y5] | 26.3 | 20 |
| LEMP (H) | 11.6 | 14.5 | Positive | 586.652 | 738.387 | [y6] | 26.3 | 20 |
| LEMP (H) | 11.6 | 14.5 | Positive | 586.652 | 738.391 | [y6] | 26.3 | 20 |
| LEMP (H) | 11.6 | 14.5 | Positive | 586.652 | 738.395 | [y6] | 26.3 | 20 |

# Supplementary Table S3. Calibration curve performance for (a) LLQV and (b) LEMP peptides.

**(a) LLQV**

|  | **CS 1** | **CS 2** | **CS 3** | **CS 4** | **CS 5** | **CS 6** | **CS 7** | **CS 8** | **CS 9** | **CS 10** |
| --- | --- | --- | --- | --- | --- | --- | --- | --- | --- | --- |
|  | **20** | **26** | **52.1** | **104** | **208** | **417** | **833** | **1670** | **2500** | **3300** |
|  | **fmol/ml** | **fmol/ml** | **fmol/ml** | **fmol/ml** | **fmol/ml** | **fmol/ml** | **fmol/ml** | **fmol/ml** | **fmol/ml** | **fmol/ml** |
|  |  |  |  |  |  |  |  |  |  |  |
| Mean | 19.5 | 26.5 | 55.4 | 108 | 214 | 408 | 827 | 1580 | 2460 | 3210 |
| SD | 1.13 | 2.3 | 3.4 | 11.3 | 15.6 | 27.0 | 55.0 | 160 | 200 | 256 |
| CV, % | 5.8 | 8.6 | 6.1 | 10.5 | 7.3 | 6.6 | 6.7 | 10.2 | 8.1 | 8.0 |
| RE, % | –2.5 | 1.9 | 6.3 | 3.8 | 2.9 | –2.2 | –0.7 | –5.4 | –1.6 | –2.7 |
| Summary table presents calibration standards above 20 fmol/ml from initial validation runs (that extended down to 7 fmol/ml) and supplemental validation runs with lowest calibration standard at 20 fmol/ml. Calibrants below 20 fmol/ml not shown. | | | | | | | | | | |

**(b) LEMP**

|  | **CS 1** | **CS 2** | **CS 3** | **CS 4** | **CS 5** | **CS 6** | **CS 7** | **CS 8** | **CS 9** | **CS 10** |
| --- | --- | --- | --- | --- | --- | --- | --- | --- | --- | --- |
|  | **20** | **26** | **52.1** | **104** | **208** | **417** | **833** | **1670** | **2500** | **3300** |
|  | **fmol/ml** | **fmol/ml** | **fmol/ml** | **fmol/ml** | **fmol/ml** | **fmol/ml** | **fmol/ml** | **fmol/ml** | **fmol/ml** | **fmol/ml** |
|  |  |  |  |  |  |  |  |  |  |  |
| Mean | 19.8 | 25.4 | 56.2 | 104 | 217 | 412 | 816 | 1590 | 2470 | 3250 |
| SD | 0.44 | 2.1 | 3.8 | 10.1 | 16.4 | 31.7 | 62.8 | 154 | 208 | 315 |
| CV, % | 2.2 | 8.2 | 6.7 | 9.8 | 7.6 | 7.7 | 7.7 | 9.6 | 8.4 | 9.7 |
| RE, % | –1.0 | –2.3 | 7.9 | 0.0 | 4.3 | –1.2 | –2.0 | –4.8 | –1.2 | –1.5 |

| Summary table presents calibration standards above 20 fmol/ml from initial validation runs (that extended down to 7 fmol/ml) and supplemental validation runs with lowest calibration standard at 20 fmol/ml. Calibrants below 20 fmol/ml not shown.  CV: coefficient of variation; LEMP: LEMPSSLMLEVPTHR; LLQV: LLQVAVEDR; RE: relative error; SD: standard deviation |
| --- |

# Supplementary Table S4. One-week freezer stability (–70⁰C) of endogenous dystrophin and mini-dystrophin in skeletal muscle lysate for (a) LLQV and (b) LEMP.

**(a) LLQV**

|  | **QCL**  **20.0 fmol/ml** | **QCM1**  **200 fmol/ml** | **QCM2**  **800 fmol/ml** | **QCH**  **2400 fmol/ml** |
| --- | --- | --- | --- | --- |
|  | 16.7 | 203 | 841 | 2630 |
|  | 16.8 | 125^a^ | 822 | 2130 |
|  | 17.0 | 146^a^ | 820 | 2620 |
|  | 16.9 | 173 | 736 | 2430 |
|  | 9.98^a^ | 193 | 570^a^ | 2250 |
|  | 17.2 | 148^a^ | 757 | 2370 |
| Mean | 15.8 | 165 | 758 | 2410 |
| SD | 2.84 | 30.1 | 101 | 199 |
| %CV | 18.0 | 18.2 | 13.3 | 8.3 |
| %RE | –21.0^b^ | –17.5 | –5.3 | 0.4 |
| n | 6 | 6 | 6 | 6 |

**(b) LEMP**

|  | **QCL**  **20.0 fmol/ml** | **QCM1**  **200 fmol/ml** | **QCM2**  **800 fmol/ml** | **QCH**  **2400 fmol/ml** |
| --- | --- | --- | --- | --- |
|  | 22.3 | 209 | 843 | 2620 |
|  | 19.0 | 132^a^ | 844 | 2150 |
|  | 16.6 | 154^a^ | 814 | 2610 |
|  | 18.8 | 176 | 744 | 2390 |
|  | 14.2^a^ | 195 | 564^a^ | 2220 |
|  | 16.8 | 158^a^ | 767 | 2430 |
| Mean | 18.0 | 171 | 763 | 2400 |
| SD | 2.76 | 28.4 | 105 | 194 |
| %CV | 15.3 | 16.6 | 13.8 | 8.1 |
| %RE | –10.0 | –14.5 | –4.6 | 0.0 |
| n | 6 | 6 | 6 | 6 |

Note: Initial QC range displayed.

CV: coefficient of variation; LLOQ: lower limit of quantification; LEMP: LEMPSSLMLEVPTHR; LLQV: LLQVAVEDR; n: number of samples; QCH: High Quality Control; QCL: Low Quality Control; QCM1: Medium Quality Control 1; QCM2: Medium Quality Control 2.; RE: relative error; SD: standard deviation

1. RE >20%
2. RE >25% (applies only to LLOQ)

# Supplementary Table S5. Normal lysate stability over a 30-week testing period following storage at –70°C.

|  | **Test Week 7 Concentration  1470 fmol/ml** | **Test Week 26 Concentration  1470 fmol/ml** | **Test Week 30 Concentration  1470 fmol/ml** | **Overall** |
| --- | --- | --- | --- | --- |
|  | 1270 | 1330 | 1510 | - |
|  | 1550 | 1330 | 1450 | - |
|  | 1380 | 1460 | 1450 | - |
|  | 1360 | 1500 | 1400 | - |
|  | 1320 | 1510 | 1020 | - |
|  | 1450 | 1490 | 1510 | - |
| Mean | 1390 | 1430 | 1390 | 1400 |
| SD | 99.5 | 92.4 | 186 | 127 |
| %CV | 7.2 | 6.5 | 13.4 | 9.0 |
| %RE | –5.4 | –2.7 | –5.4 | –6.0 |
| n | 6 | 6 | 6 | 18 |

Stability is demonstrated if the mean concentration of the stored QC samples is within ± 20% compared to their nominal concentration with a CV ≤20%.

CV: coefficient of variation; QC: quality control; RE: relative error; SD: standard deviation

# Supplementary Table S6. Stability of mini-dystrophin in skeletal muscle lysate after cycles of freezing and thawing for (a) LLQV and (b) LEMP.

**(a) LLQV**

|  | **FT1-QCL  20.0 fmol/mL** | **FT2-QCL**  **20.0 fmol/mL** | **FT3-QCL**  **20.0 fmol/mL** | **FT1-QCH**  **2400 fmol/mL** | **FT2-QCH**  **2400 fmol/mL** | **FT3-QCH**  **2400 fmol/mL** |
| --- | --- | --- | --- | --- | --- | --- |
|  | 79.5b | 17.8 | 16.5 | 2410 | 2360 | 2300 |
|  | 23.3 | 17.3 | 7.55a | 2230 | 2340 | 2380 |
|  | 18.1 | 16.9 | 13.0a | 2120 | 1990 | 2240 |
|  | 16.8 | 13.2a | 18.0 | 2380 | 2320 | 2340 |
|  | 14.1a | 17.3 | 90.3b | 2530 | 2640 | 2510 |
|  | 16.6 | 17.1 | 15.9a | 2580 | 2270 | 2490 |
| Mean | 17.8 | 16.6 | 14.2 | 2380 | 2320 | 2380 |
| SD | 3.41 | 1.69 | 4.13 | 175 | 208 | 106 |
| %CV | 19.2 | 10.2 | 29.1 | 7.4 | 9.0 | 4.5 |
| %RE | –11.0 | –17.0 | –29.0 | –0.8 | –3.3 | –0.8 |
| n | 5 | 6 | 5 | 6 | 6 | 6 |

**(b) LEMP**

|  | **FT1-QCL 20.0**  **fmol/ml** | **FT2-QCL**  **20.0**  **fmol/ml** | **FT3-QCL**  **20.0**  **fmol/ml** | **FT1-QCH**  **2400**  **fmol/ml** | **FT2-QCH**  **2400**  **fmol/ml** | **FT3-QCH**  **2400**  **fmol/ml** |
| --- | --- | --- | --- | --- | --- | --- |
|  | 67.7b | 15.0a | 15.9a | 2310 | 2170 | 2190 |
|  | 20.2 | 17.9 | 13.1a | 2220 | 1930 | 2300 |
|  | 21.8 | 17.5 | 16.3 | 1950 | 1890a | 2150 |
|  | 18.8 | 17.7 | 18.7 | 2350 | 2280 | 2370 |
|  | 16.5 | 17.5 | 81.1b | 2540 | 2460 | 2550 |
|  | 18.8 | 18.6 | 17.4 | 2490 | 2210 | 2330 |
| Mean | 19.2 | 17.4 | 16.3 | 2310 | 2160 | 2320 |
| SD | 1.96 | 1.23 | 2.08 | 212 | 216 | 143 |
| %CV | 10.2 | 7.1 | 12.8 | 9.2 | 10.0 | 6.2 |
| %RE | –4.0 | –13.0 | –18.5 | –3.8 | –10.0 | –3.3 |
| n | 5 | 6 | 5 | 6 | 6 | 6 |

Note: Freeze-Thaw is acceptable for up to 2 cycles.

CV: coefficient of variation; FT: freeze/thaw cycle; LEMP: LEMPSSLMLEVPTHR; LLQV: LLQVAVEDR; n: number of samples; QCH: high quality control; QCL: low quality control; RE: relative error; SD: standard deviation.

a. RE >20%.

b. Outlier based on Dixon Test (99% confidence).

# Supplementary Table S7. Dystrophin and total protein analysis for (a) control, non-dystrophic subjects, (b) BMD patients and (c) DMD patients.

**(a) Controls, non-dystrophic subjects**

| **Anonymized Subject, #** | **LCMS Dystrophin Concentration, fmol/mL** | **BCA Total Protein Concentration, mg/mL** | **Dystrophin Tissue Concentration, fmol/mg** | **Average Relative to Control Group, %** |
| --- | --- | --- | --- | --- |

| Control 1 | 1110 | 0.391 | 2838.9 | 82.5 |
| --- | --- | --- | --- | --- |
| Control 2 | 2360 | 0.789 | 2991.1 | 86.9 |
| Control 3 | 1620 | 0.581 | 2788.3 | 81.1 |
| Control 4 | 1240 | 0.486 | 2551.4 | 74.2 |
| Control 5 | 2380 | 0.657 | 3622.5 | 105.3 |
| Control 6 | 3500 | 1.29 | 2713.2 | 78.9 |
| Control 7 | 3230 | 0.631 | 5118.9 | 148.8 |
| Control 8 | 2000 | 0.677 | 2954.2 | 85.9 |
| Control 9 | 2130 | 0.602 | 3538.2 | 102.9 |
| Control 10 | 2800 | 0.607 | 4612.9 | 134.1 |
| Control 11 | 2170 | 0.841 | 2580.3 | 75.0 |
| Control 12 | 1930 | 0.505 | 3821.8 | 111.1 |
| Control 13 | 2070 | 0.474 | 4367.1 | 126.9 |
| Control 14 | 772 | 0.27 | 2859.3 | 83.1 |
| Control 15 | 2880 | 0.983 | 2929.8 | 85.2 |
| Control 16 | 2620 | 0.737 | 3555.0 | 103.3 |
| Control 17 | 3210 | 0.629 | 5103.3 | 148.3 |
| Control 18 | 2470 | 0.724 | 3411.6 | 99.2 |
| Control 19 | 2520 | 0.598 | 4214.0 | 122.5 |
| Control 20 | 1350 | 0.605 | 2231.4 | 64.9 |
|  | **Average, %** | **Average, fmol/ml** | **SD** | **CV, %** |
| **Overall** | 100.0 | 3440.2 | 857.2 | 24.9 |

*continued*

**(b) BMD patients**

| **Anonymized Subject, #** | **LCMS Dystrophin Concentration, fmol/ml** | **BCA Total Protein Concentration, mg/ml** | **Dystrophin Tissue Concentration, fmol/mg** | **Average Relative to Control Group, %** |
| --- | --- | --- | --- | --- |
| BMD 21 | 436 | 0.334 | 1305.4 | 37.9 |
| BMD 22 | 952 | 0.577 | 1649.9 | 48.0 |
| BMD 23 | 2430 | 0.836 | 2906.7 | 84.5 |
| BMD 24 | 1760 | 0.634 | 2776.0 | 80.7 |
| BMD 25 | 497 | 0.593 | 838.1 | 24.4 |
| BMD 26 | 72.1 | 0.473 | 152.4 | 4.4 |
| BMD 27 | 185 | 0.428 | 432.2 | 12.6 |
| BMD 28 | 115 | 0.36 | 319.4 | 9.3 |
| BMD 29 | 436 | 0.569 | 766.3 | 22.3 |
| BMD 30 | 55.5 | 0.36 | 154.2 | 4.5 |
| BMD 31 | 114 | 0.821 | 138.9 | 4.0 |
| BMD 32 | 788 | 1.3 | 606.2 | 17.6 |
| BMD 33 | 2130 | 0.78 | 2730.8 | 79.4 |
| BMD 34 | 1450 | 1.35 | 1074.1 | 31.2 |
| BMD 35 | 523 | 0.663 | 788.8 | 22.9 |
| BMD 36 | 1280 | 1.31 | 977.1 | 28.4 |
| BMD 37 | 2270 | 1.69 | 1343.2 | 39.0 |
| BMD 38 | 615 | 0.823 | 747.3 | 21.7 |
| BMD 39 | 918 | 0.96 | 956.3 | 27.8 |
| BMD 40 | 1000 | 0.891 | 1122.3 | 32.6 |
|  | **Average, %** | **Average, fmol/ml** | **SD** | **CV, %** |
| **Overall** | 31.7 | 1089.3 | 845.7 | 77.6 |

*continued*

**(c) DMD patients**

| **Anonymized Subject, #** | **LCMS Dystrophin Concentration, fmol/ml** | **BCA Total Protein Concentration, mg/ml** | **Dystrophin Tissue Concentration, fmol/mg** | **Average Relative to Control Group, %** |
| --- | --- | --- | --- | --- |

| DMD 41 | 10a | 0.332 | 30.1 | 0.9 |
| --- | --- | --- | --- | --- |
| DMD 42 | 44.7 | 0.617 | 72.4 | 2.1 |
| DMD 43 | 10a | 0.689 | 14.5 | 0.4 |
| DMD 44 | 70.9 | 0.477 | 148.6 | 4.3 |
| DMD 45 | 10a | 0.349 | 28.7 | 0.8 |
| DMD 46 | 268 | 0.973 | 275.4 | 8.0 |
| DMD 47 | 338 | 0.782 | 432.2 | 12.6 |
| DMD 48 | 49.6 | 0.643 | 77.1 | 2.2 |
| DMD 49 | 57.7 | 0.665 | 86.8 | 2.5 |
| DMD 50 | 105 | 0.598 | 175.6 | 5.1 |
| DMD 51 | 10a | 0.414 | 24.2 | 0.7 |
| DMD 52 | 10a | 0.544 | 18.4 | 0.5 |
| DMD 53 | 471 | 0.568 | 829.2 | 24.1 |
| DMD 54 | 10a | 0.374 | 26.7 | 0.8 |
| DMD 55 | 24.1 | 0.349 | 69.1 | 2.0 |
| DMD 56 | 24.1 | 0.56 | 43.0 | 1.3 |
| DMD 57 | 10a | 0.626 | 16.0 | 0.5 |
| DMD 58 | 221 | 0.808 | 273.5 | 8.0 |
| DMD 59 | 106 | 0.422 | 251.2 | 7.3 |
| DMD 60 | 555 | 0.673 | 824.7 | 24.0 |
|  | **Average, %** | **Average, fmol/ml** | **SD** | **CV, %** |
| **Overall** | 5.4 | 185.9 | 247.0 | 132.9 |

BCA: bicinchoninic assay; BMD: Becker muscular dystrophy; CV: coefficient of variation; DMD: Duchenne muscular dystrophy; LCMS: liquid chromatography–mass spectrometry; SD: standard deviation.

a. Below limit of quantification (20.0 fmol/ml). Concentrations below the limit of quantification were imputed as 0.5*LLOQ for statistical analyses.

# Supplementary Table S8. SILAC mini-dystrophin amino acid sequence. All leucine residues were labelled with 13C. Peptide sequences of interest are underlined. Amino acids in bold are labelled on target peptide sequences.

| **SILAC Mini-Dystrophin Amino Acid Sequence** |
| --- |
| MLWWEEVEDCYEREDVQKKTFTKWVNAQFSKFGKQHIENLFSDLQDGRRLLDLLEGLTGQ  KLPKEKGSTRVHALNNVNKALRVLQNNNVDLVNIGSTDIVDGNHKLTLGLIWNIILHWQV  KNVMKNIMAGLQQTNSEKILLSWVRQSTRNYPQVNVINFTTSWSDGLALNALIHSHRPDL  FDWNSVVCQQSATQRLEHAFNIARYQLGIEKLLDPEDVDTTYPDKKSILMYITSLFQVLP  QQVSIEAIQEVEMLPRPPKVTKEEHFQLHHQMHYSQQITVSLAQGYERTSSPKPRFKSYA  YTQAAYVTTSDPTRSPFPSQHLEAPEDKSFGSSLMESEVNLDRYQTALEEVLSWLLSAED  TLQAQGEISNDVEVVKDQFHTHEGYMMDLTAHQGRVGNILQLGSKLIGTGKLSEDEETEV  QEQMNLLNSRWECLRVASMEKQSNLHRVLMDLQNQKLKELNDWLTKTEERTRKMEEEPLG  PDLEDLKRQVQQHKVLQEDLEQEQVRVNSLTHMVVVVDESSGDHATAALEEQLKVLGDRW  ANICRWTEDRWVLLQDQPDLAPGLTTIGASPTQTVTLVTQPVVTKETAISK**L**EMPSS**L**M**L**  EVPTHRLLQQFPLDLEKFLAWLTEAETTANVLQDATRKERLLEDSKGVKELMKQWQDLQG  EIEAHTDVYHNLDENSQKILRS**L**EGSDDAV**LL**QRRLDNMNFKWSELRKKSLNIRSHLEAS  SDQWKRLHLSLQELLVWLQLKDDELSRQAPIGGDFPAVQKQNDVHRAFKRELKTKEPVIM  STLETVRIFLTEQPLEGLEKLYQEPRELPPEERAQNVTRLLRKQAEEVNTEWEKLNLHSA  DWQRKIDETLERLQELQEATDELDLKLRQAEVIKGSWQPVGDLLIDSLQDHLEKVKALRG  EIAPLKENVSHVNDLARQLTTLGIQLSPYNLSTLEDLNTRWK**LL**QVAVEDRVRQLHEAHR  DFGPASQHFLSTSVQGPWERAISPNKVPYYINHETQTTCWDHPKMTELYQSLADLNNVRF  SAYRTAMKLRRLQKALCLDLLSLSAACDALDQHNLKQNDQPMDILQIINCLTTIYDRLEQ  EHNNLVNVPLCVDMCLNWLLNVYDTGRTGRIRVLSFKTGIISLCKAHLEDKYRYLFKQVA  SSTGFCDQRRLGLLLHDSIQIPRQLGEVASFGGSNIEPSVRSCFQFANNKPEIEAALFLD  WMRLEPQSMVWLPVLHRVAAAETAKHQAKCNICKECPIIGFRYRSLKHFNYDICQSCFFS  GRVAKGHKMHYPMVEYCTPTTSGEDVRDFAKVLKNKFRTKRYFAKHPRMGYLPVQTVLEG  DNMET |

For SILAC mini-dystrophin generation, the mini-dys-FLAG expression vector was transfected into HEK-293 cells cultured in SILAC 13C-L-leucine medium containing 10% dialyzed foetal bovine serum. The harvested cells were lysed with NP40, and the clarified lysate was purified over a FLAG affinity column. The final formulation buffer of the purified protein was Tris Buffered Saline, pH 7.2–7.4. Purified protein was characterized by SDS-PAGE, Western blot, and analysed for endotoxin level. Concentration was determined by BCA using bovine serum albumin as the standard.

BCA: bicinchoninic assay; BSA: bovine serum albumin.

# Supplementary Figures

## Supplementary Fig. S1.


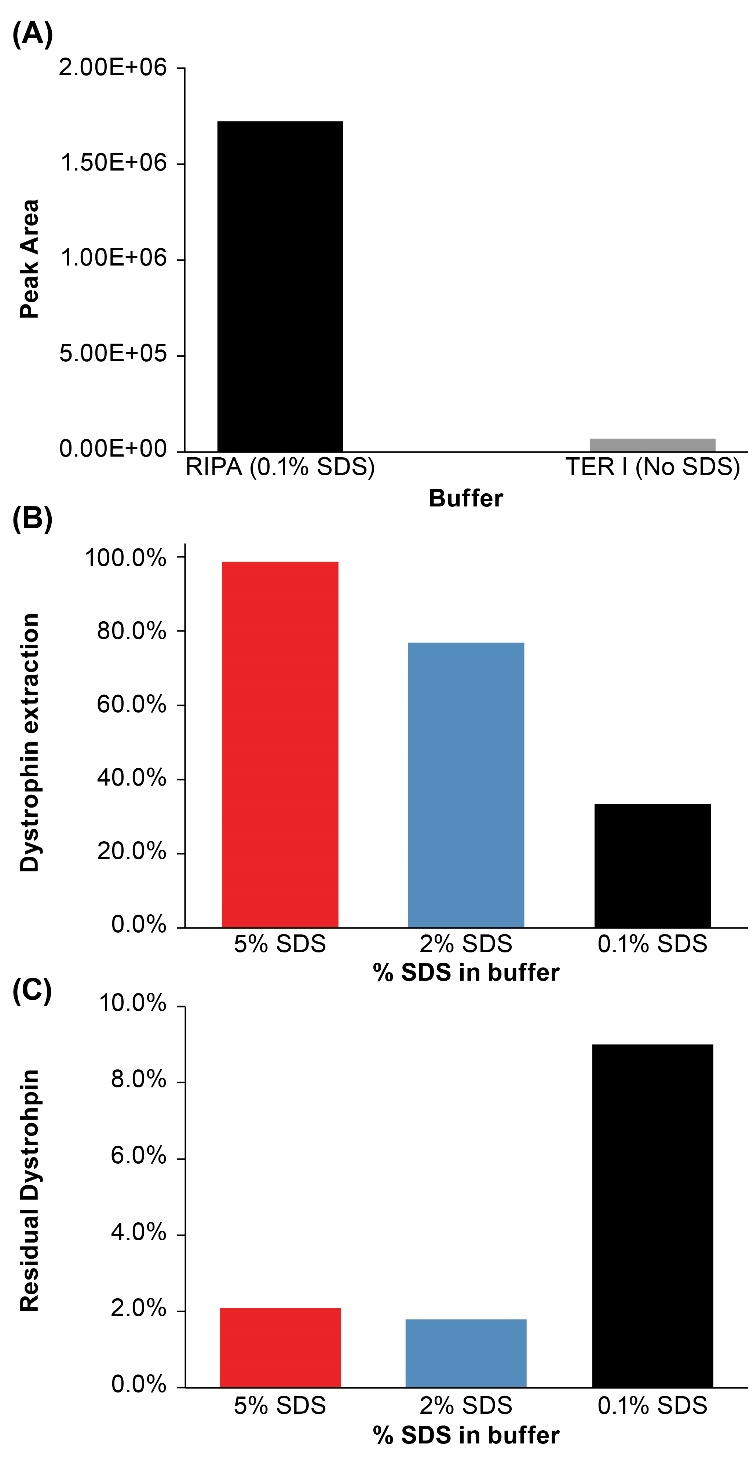
The effect of sodium dodecyl sulfate (SDS) on percentage dystrophin extraction from equal weights of normal human muscle tissue**.** (**a**) RIPA (0.1% SDS) vs. TER-I (no SDS); (**b**) effect of SDS (5% vs. 2%, vs. 0.1%) on percentage dystrophin extraction; (**c**) percentage of dystrophin re-extraction from debris relative to 5% SDS. SDS: sodium dodecyl sulfate (10% vs. 5%).

##
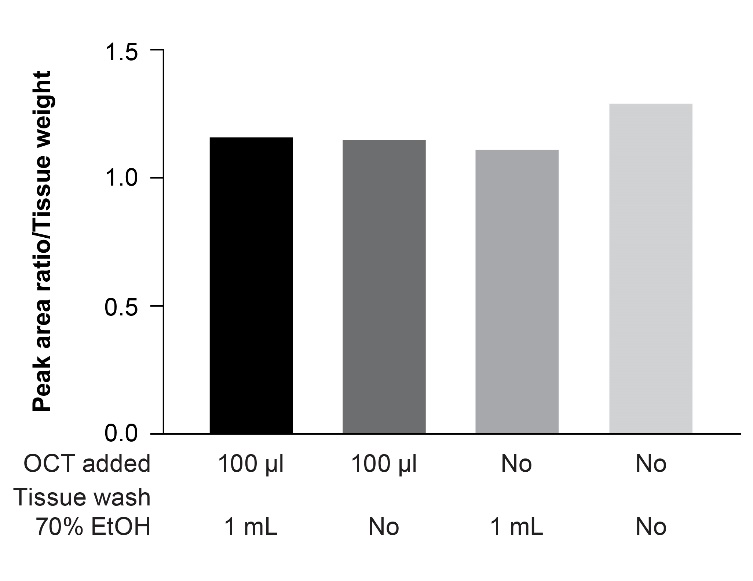
Supplementary Fig. S2.

The effect of OCT on assay performance as assessed in normal human tissue. Weighted muscle tissues with and without OCT were washed with 1 ml of 70% ethanol and results were compared to the no-wash samples.

EtOH: ethanol alcohol; OCT: optimal cutting temperature.

## Supplementary Fig. S3.

Correlation of the dystrophin quantification data calculated from LLQV and SLEG peptides. The R square values are calculated separately for Normal, BMD and DMD sample sets and show good agreement between the two peptides. Trendline fittings are linear from 0 intercept.


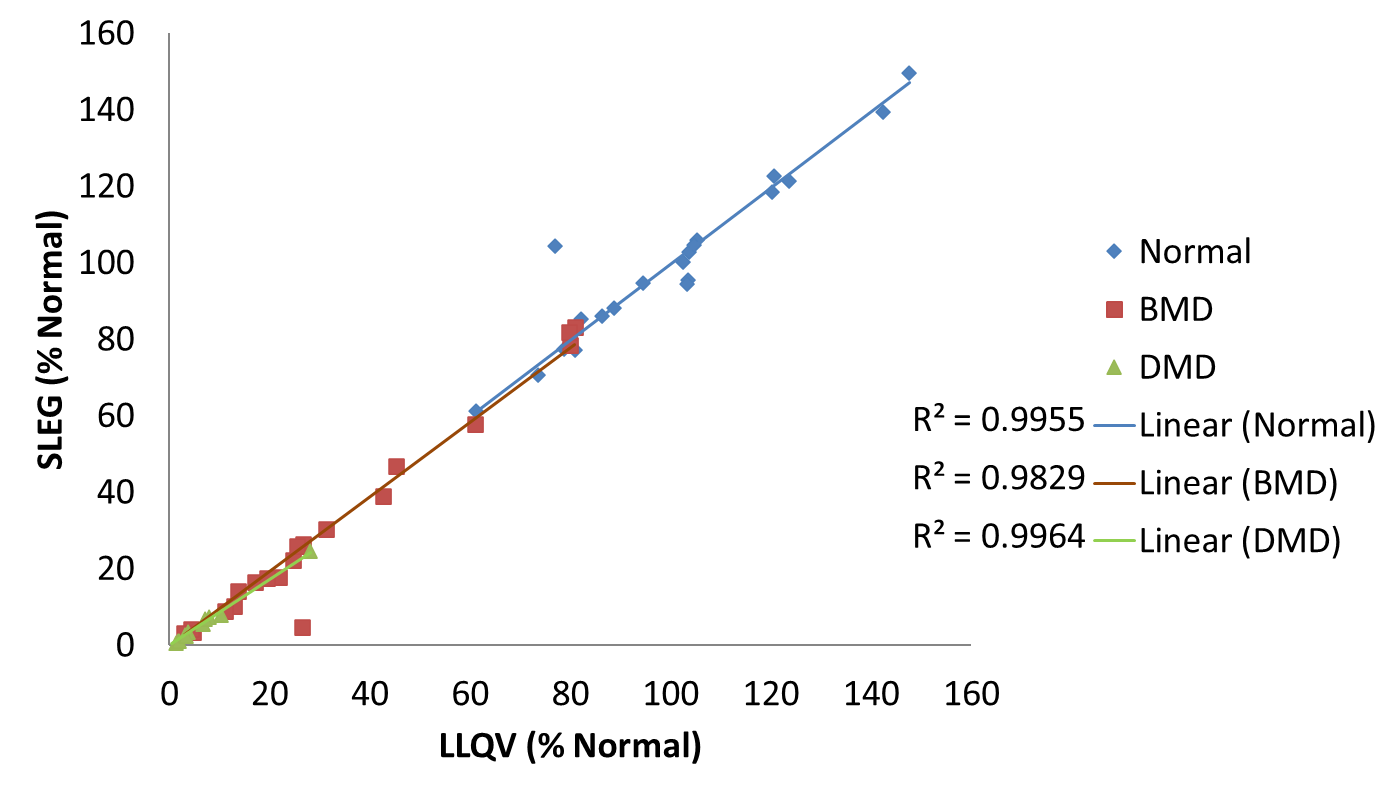


## Supplementary Fig. S4.

Representative LLQV peptide extracted ion chromatograms from age-matched normal, BMD and DMD muscle biopsies. (a) Normal sample 9, 102.9% of mean normal; (**b**) BMD sample 34, 31.2% of mean normal; (**c**) DMD sample 50, 5.1% of mean normal; (d) Normal sample 16, 103.3% of mean normal; (**e**) BMD sample 40, 32.6% of mean normal; (**f**) DMD sample 59, 7.3% of mean normal.


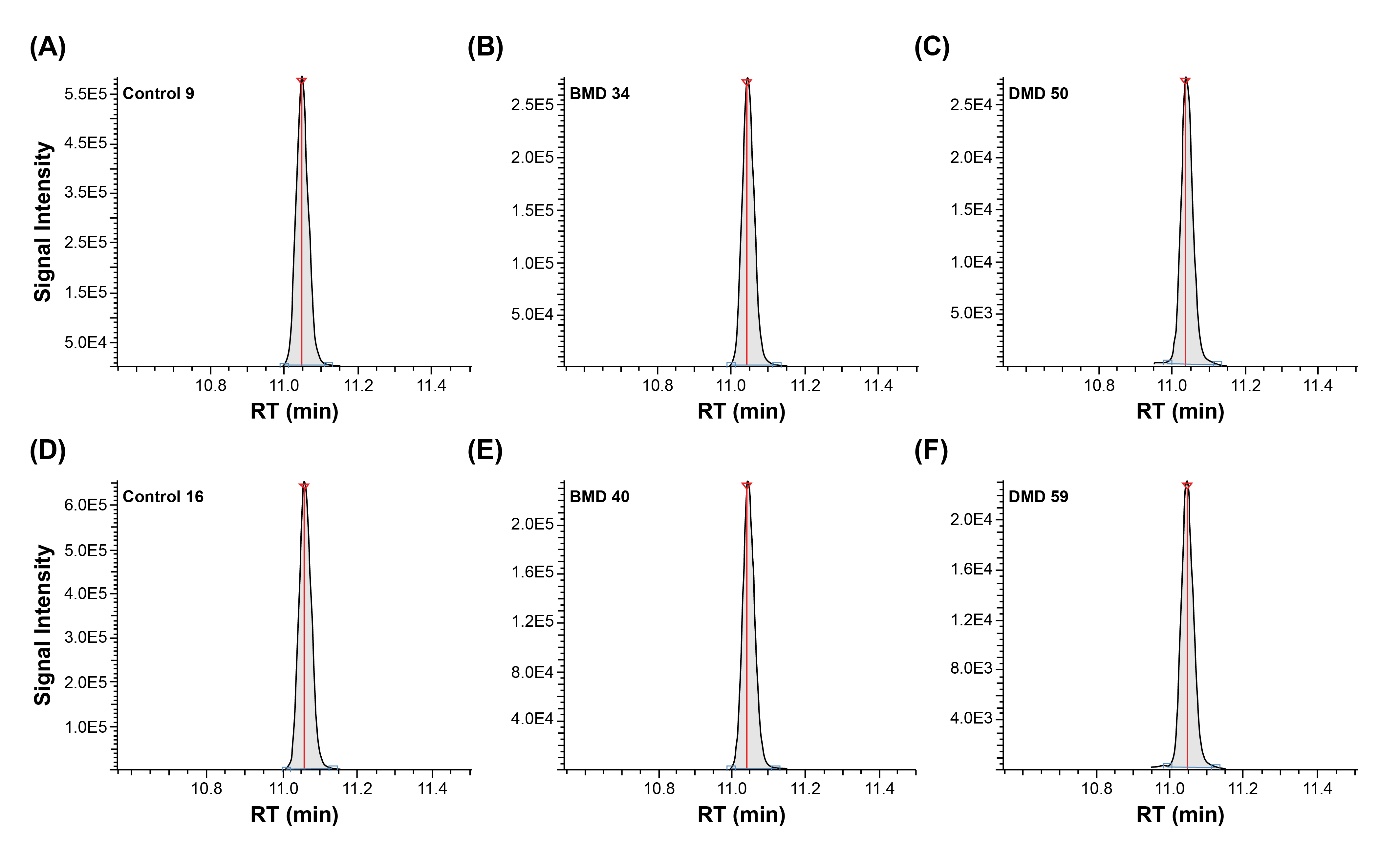


BMD: Becker muscular dystrophy; DMD: Duchenne muscular dystrophy; LLQV: LLQVAVEDR

## Supplementary Fig. S5.

(**a**) Expression of dystrophin in paediatric males and females, normal population; (**b**) total protein content in age-matched normal, BMD and DMD muscle biopsies.


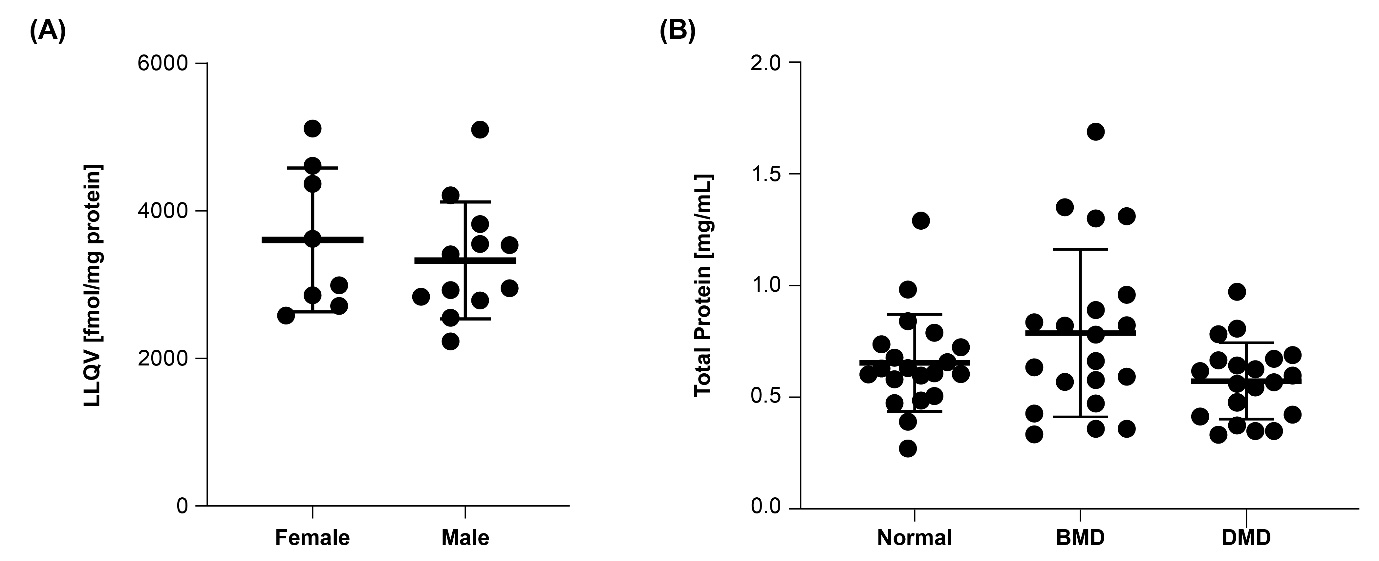


BMD: Becker muscular dystrophy; DMD: Duchenne muscular dystrophy; LLQV: LLQVAVEDR
